# Seasonal shift in epidemics of respiratory syncytial virus infection in Japan

**DOI:** 10.1017/S0950268821000340

**Published:** 2021-02-11

**Authors:** Takeshi Miyama, Nobuhiro Iritani, Takayuki Nishio, Tomohiko Ukai, Yuka Satsuki, Hiromi Miyata, Ayumi Shintani, Satoshi Hiroi, Kazushi Motomura, Kazuo Kobayashi

**Affiliations:** 1 Department of Public Health, Osaka Institute of Public Health, Osaka, Japan; 2 Department of Social Medicine, Osaka University Graduate School of Medicine, Suita, Japan; 3 Department of Medical Statistics, Osaka City University Graduate School of Medicine, Osaka, Japan; 4 Department of Microbiology, Osaka Institute of Public Health, Osaka, Japan

**Keywords:** Epidemics, respiratory syncytial virus infections, seasonal shift

## Abstract

In Japan, respiratory syncytial virus (RSV) infection generally has occurred during autumn and winter. However, a possible change in the seasonal trend of RSV infection has been observed recently. The current study was conducted to determine whether the epidemic season of RSV infection in Japan has indeed changed significantly. We used expectation-based Poisson scan statistics to detect periods with high weekly reported RSV cases (epidemic cluster), and the epidemic clusters were detected between September and December in the 2012–2016 seasons while those were detected between July and October in the 2017–2019 seasons. Non-linear and linear ordinary least squares regression models were built to evaluate whether there is a difference in year trend in the epidemic seasonality, and the epidemic season was shifted to earlier in the year in 2017–2019 compared to that in 2012–2016. Although the reason for the shift is unclear, this information may help in clinical practice and public health.

Respiratory syncytial virus (RSV) infection can cause bronchiolitis and pneumonia, especially in infants, older adults and high-risk populations, including individuals with immunodeficiency and/or congenital heart disease. For the high-risk infants and children, palivizumab, a humanised monoclonal antibody against RSV F glycoprotein, use is covered by national health insurance in Japan to prevent the development of severe conditions. Appropriate planning for palivizumab administration is important for the prevention of serious RSV disease in high-risk infants and children because it should be given before the epidemic. Recently, a possible change in the seasonal trend of RSV infection in Japan has been observed [1] but not statistically analysed. The objective of the current study was to use statistical analyses to determine whether the epidemic season of RSV infection has indeed changed significantly.

In accordance with the Infectious Disease Surveillance Control Law of Japan, RSV infection was added to the list of category V infectious diseases. RSV is reported as part of the paediatric sentinel surveillance system under the National Epidemiological Surveillance of Infectious Diseases (NESID) Program; such reports are based on laboratory diagnosis of specimens (nasal/throat swabs) using enzyme immunoassays to detect RSV antigen and/or nucleic acid amplification tests for the corresponding RSV gene. Paediatric sentinel sites include 3000 hospitals and clinics. The national health insurance coverage of the use of the RSV antigen detection assay has been expanded from inpatient use to infant outpatients or outpatients for whom palivizumab is indicated since October 2011 [1]. Based on the establishment of laboratory diagnostic tests after the year of 2012 (the timing of the expansion of the insurance coverage for the test as mentioned above), our study analysed NESID data from Infectious Diseases Weekly Report (IDWR) [2] for the years 2012–2019.

For analyses, we used expectation-based Poisson scan statistics [3] to detect periods with high weekly reported RSV cases (epidemic clusters) for the non-spatial time series data. An interval of 15 weeks or less that contained the largest epidemic cluster of RSV infection was defined (e.g. in the 2013 season, weeks 38–52 (16 September–29 December) was identified as the epidemic cluster, Fig. 1a). The length of 15 weeks, which is shorter than the usual RSV season, was subjectively chosen. A new dataset was subsequently formed to include the weeks of the identified epidemic clusters in the years 2012–2019. The analysis of the longer period, the years 2006–2019 is available in the Supplementary material (to investigate the sensitivity of the study term).

**Fig. 1.**
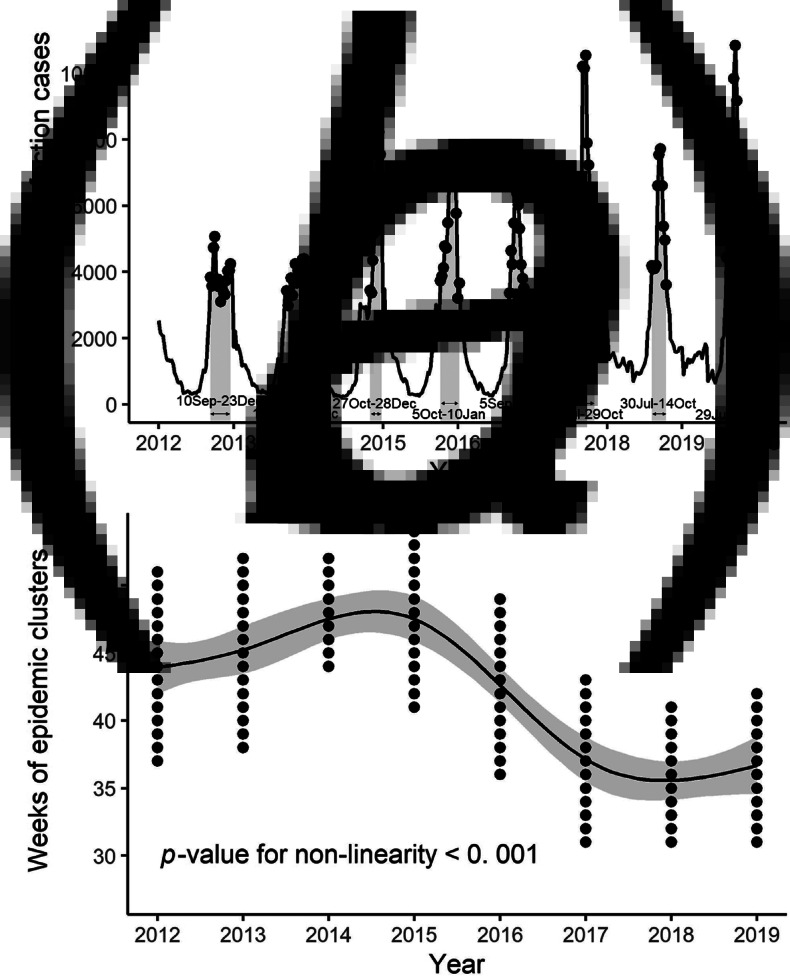
Weeks of disease clusters per year detected by the scan statistics, and the predictions of year effect on the weeks of the clusters in 2012–2019, as obtained from the non-linear OLS regression models. (a) The number of weekly reported RSV infection cases from paediatric sentinel sites (solid lines) and the weeks of detected disease clusters (dots and grey areas). The dates in the plot are the cluster-start/end-points. (b) The prediction (solid line) from the non-linear OLS regression model for the weeks of the clusters (dots).

A non-linear ordinary least squares (OLS) regression model using cubic spline with 5 degrees of freedom was constructed with the use of the new dataset to estimate the effect of each year on the weeks of RSV infection clusters. In order to evaluate whether there is a difference in year trend in the epidemic seasonality between the 2012–2016 and 2017–2019 seasons, the year and the period variables (i.e. a dummy variable for the two periods) was added to the regression model in a linear OLS regression model. The two groups above (the 2012–2016 and 2017–2019 seasons) were defined empirically: the cluster of the first group started and finished later (September/October–December/January) compared to the second group (July–October). All statistical analyses were performed using R, version 3.6.1 [4].

The weeks of the epidemic clusters of RSV infection detected per season are plotted in Figure 1a. The weeks of the clusters of the 2012–2016 seasons were weeks 37–51 (10 September–23 December), 38–52 (16 September–29 December), 44–52 (27 October–28 December), 41–1 (5 October–10 January) of the next year and 36–49 (5 September–11 December), respectively. In contrast, those of the 2017–2019 seasons were weeks 31–43 (31 July–29 October), 31–41 (30 July–14 October) and 31–42 (29 July–20 October), respectively. The weeks of the clusters of the 2006–2011 seasons were close to or earlier than those of the 2012–2016 seasons (see Fig. S1).

The non-linear OLS regression model indicated that the year effect on the weeks of epidemic clusters was statistically significant (*P*-value for year effect and non-linearity were both <0.001, Fig. 1b); thus, the epidemic season has changed. Using the linear OLS model, the effect of the defined periods (2012–2016 and 2017–2019 seasons) was statistically significant (*P* < 0.001). Thus, the epidemic season was shifted to earlier in the year in 2017–2019 compared to that in 2012–2016. This result demonstrated that the epidemic season of RSV infection in Japan shifted from autumn and winter to summer and autumn since 2017. The backgrounds (age and sex) of patients with RSV infection were similar throughout the study period. Similarly, there was no meaningful difference in clinical manifestations reported. Notably, no major change in viral genetics (mutations and genotypes of RSV) has been reported during this period.

In Japan, RSV infection generally has occurred during autumn and winter. However, the epidemic season of RSV infection in Japan has shifted to summer and autumn since 2017. We have found a similar tendency in the different regions in Japan except for Okinawa Prefecture, a subtropical area (data not shown). In the United States and other areas with similar climates, RSV infection occurs during autumn, winter and spring [5, 6]. Outside of the United States, RSV infection usually peaks during wet months in areas with high annual precipitation and during cooler months in hot and dry areas [7]. Thus, the epidemics of RSV infection may be associated with environmental/climatic factors. Similarly, a seasonal change in RSV infection epidemics since 2007 was reported in Brazil; the change represented a shift of approximately 1 month earlier was seen in the period 1999–2006 [8]. As shown in the current study, the weeks of epidemic clusters in Japan have shifted 8 weeks earlier (the difference of the epidemic cluster weeks between 2012–2016 and 2017–2019 from the linear OLS regression model), although the precise reasons for this seasonal shift remain undefined. In Japan, the number of live births is stable for a decade, while visitors from abroad dramatically increased (about 8 million visitors in 2012, 32 million in 2019). The peak of daily rainfall and mean relative humidity have shown a slight increase recently. Future studies are needed to clarify the factors influencing the number of RSV disease.

The current study used statistical analyses to demonstrate a seasonal shift from autumn and winter to summer and autumn since 2017 in RSV infection epidemics in Japan. This analysis may improve our understanding of current epidemics of RSV infection and facilitate the timing of administration of a monoclonal antibody against RSV, palivizumab, to prevent severe outcomes from RSV infection in high-risk groups.

## Data Availability

The data used in this study are available from the National Institute of Infectious Diseases, Infectious Diseases Weekly Report (IDWR) at: https://www.niid.go.jp/niid/en/idwr-e.html.

## Supplementary material

For supplementary material accompanying this paper visit http://dx.doi.org/10.1017/S0950268821000340. click here to view supplementary material
