# Supplementary material for: Seasonal shift in epidemics of respiratory syncytial virus infection in Japan
Source: Epidemiol Infect. 2021 Feb 11;149:e55. doi: 10.1017/S0950268821000340 (PMC8060823; doi:10.1017/S0950268821000340)
Supplement: Supplementary file 1 [file S0950268821000340sup001.docx]

**Supplementary material for: “Seasonal Shift in Epidemics of Respiratory Syncytial Virus Infection in Japan”**

Takeshi Miyama, Nobuhiro Iritani, Takayuki Nishio, Tomohiko Ukai, Yuka Satsuki, Hiromi Miyata, Ayumi Shintani, Satoshi Hiroi, Kazushi Motomura, Kazuo Kobayashi

# **Sensitivity analysis**

To evaluate the sensitivity of the study period, the longer period, the years 2006–2019 (vs 2012–2019 in the main analysis) of the respiratory syncytial virus (RSV) infection surveillance data were investigated although the reporting rate might be varied in this period due to the insurance coverage change. Expectation-based Poisson scan statistics (the same method described in the main text) was used to detect periods with high weekly reported RSV cases (epidemic clusters). This period was chosen because the insurance coverage for the test was expanded in 2006 from inpatients who are less than 3 years old to all inpatients. An interval of 15 weeks or less that contained the largest epidemic cluster of RSV infection was defined. A new dataset was subsequently formed to include the weeks of the identified epidemic clusters in the years 2006–2019. A non-linear ordinary least squares (OLS) regression model using cubic spline with 7 degrees of freedom was constructed with the use of the new dataset to estimate the effect of each year on the weeks of RSV infection clusters.

# **Results and discussion**

The weeks of the epidemic clusters of RSV infection detected per season are plotted in Figure S1. The weeks of the clusters of the 2006–2011 seasons were Weeks 50–6 (11 December–11 February) of the next year, 49–52 (3 December––30 December), 46–52 (10 November–28 December), 51–9 (14 December–7 March), 48–1 (29 November–9 January), and 47–1 (21 November–8 January), respectively (Figure S1). The weeks of the clusters of the 2012–2019 seasons were the same results as the main analysis. The non-linear OLS regression model indicated that the year effect on the weeks of epidemic clusters was statistically significant (*p*-value for year effect and non-linearity were both < 0.001, Figure S2). The decreasing trend of the weeks of the epidemic clusters detected as time elapses in this sensitivity analysis was consistent to that in the original analysis (using the shorter period, the 2012–2019 seasons).


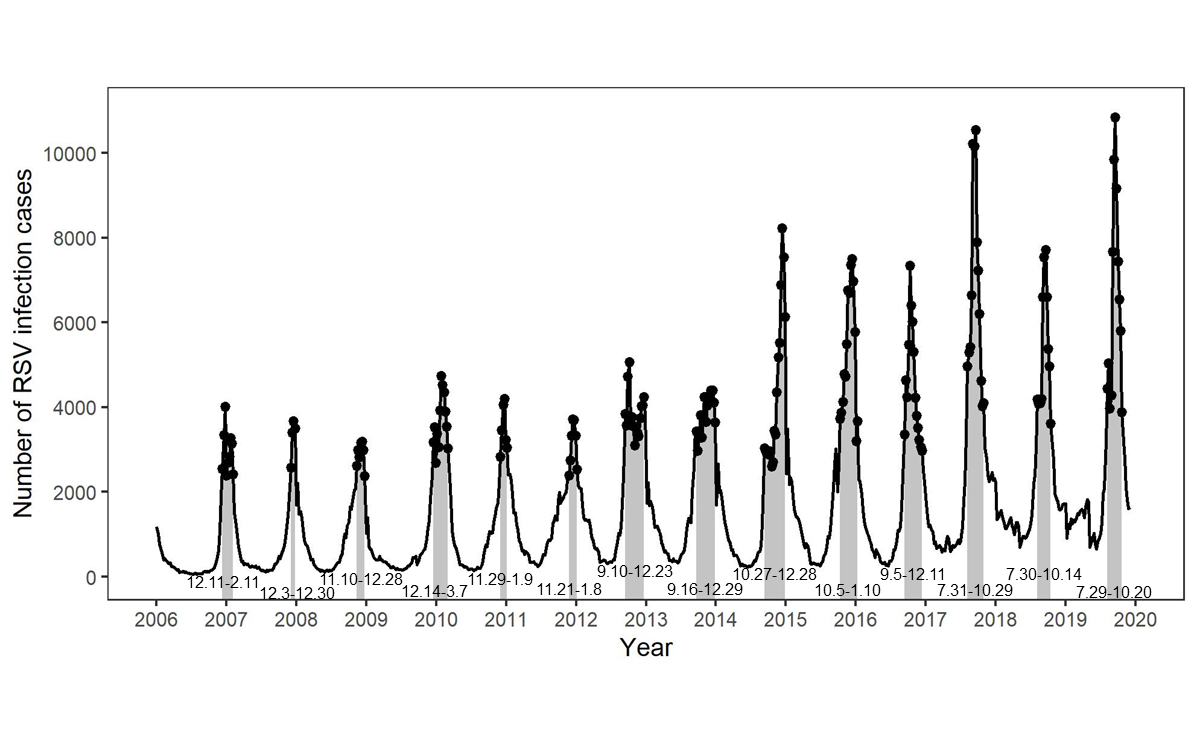
**Figure S1.** Disease clusters per year detected by the scan statistics. The solid line shows the number of weekly reported Respiratory Syncytial Virus (RSV) infection cases from pediatric sentinel sites. The dots and grey areas show the periods of detected disease clusters. The dates in the plot are the cluster-start/end-points.


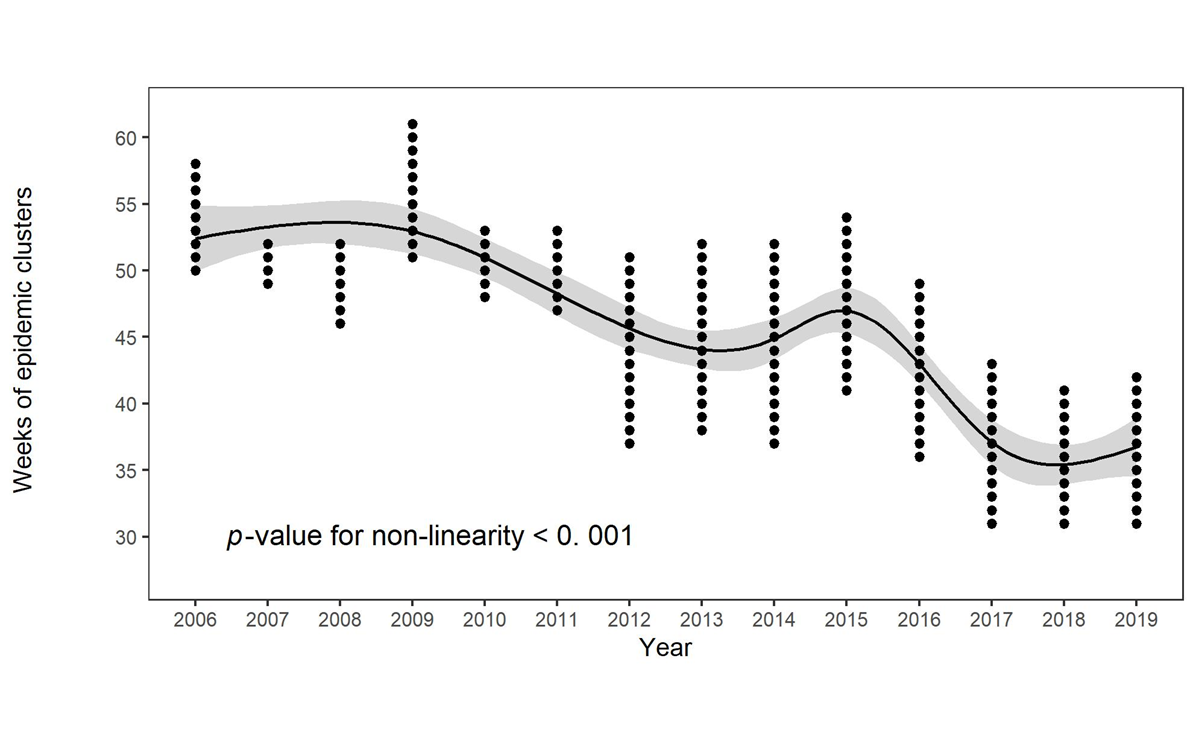
**Figure S2.** The predictions of year effect on the weeks of the clusters in 2006–2019, as obtained from the non-linear ordinary least squares (OLS) regression model. The solid line and grey area show the prediction and its 95% confidence interval from the non-linear OLS regression model. The dots show the weeks of the clusters. Weeks over 53 (y-axis) indicates the weeks of the next year
